# A State Optimization Model Based on Kalman Filtering and Robust Estimation Theory for Fusion of Multi-Source Information in Highly Non-linear Systems

**DOI:** 10.3390/s19071687

**Published:** 2019-04-09

**Authors:** Muhammad Adeel Akram, Peilin Liu, Muhammad Owais Tahir, Waqas Ali, Yuze Wang

**Affiliations:** 1 Shanghai Key Laboratory of Navigation and Location-Based Services, School of Electronic Information and Electrical Engineering, Shanghai Jiao Tong University, Shanghai 200240, China; adeelakram03@gmail.com (M.A.A.); owais_tahir@sjtu.edu.cn (M.O.T.); vaqas11@sjtu.edu.cn (W.A.); wangyuze90@163.com (Y.W.); 2 Department of Electrical and Computer Engineering, COMSATS University Islamabad, Wah Campus, GT Road, Wah Cantt. 47040, Pakistan

**Keywords:** EKF (Extended Kalman Filter), IEKF (Iterative Extended Kalmna Filter), iterative Kalman filter, reweighted least square, IRWLS (Iterative Reweighted Least Square), multi-sensor integration, robust filtering, robust estimation, non-linear system

## Abstract

Consistent state estimation is a vital requirement in numerous real life applications from localization to multi-source information fusion. The Kalman filter and its variants have been successfully used for solving state estimation problems. Kalman filtering-based estimators are dependent upon system model assumptions. A deviation from defined assumptions may lead to divergence or failure of the system. In this work, we propose a Kalman filtering-based robust state estimation model using statistical estimation theory. Its primary intention is for multiple source information fusion, although it is applicable to most non-linear systems. First, we propose a robust state prediction model to maintain state constancy over time. Secondly, we derive an error covariance estimation model to accept deviations in the system error assumptions. Afterward, an optimal state is attained in an iterative process using system observations. A modified robust MM estimation model is executed within every iteration to minimize the impact of outlying observation and approximation errors by reducing their weights. For systems having a large number of observations, a subsampling process is introduced to intensify the optimized solution redundancy. Performance is evaluated for numerical simulation and real multi sensor data. Results show high precision and robustness of proposed scheme in state estimation.

## 1. Introduction

Reliable state estimation from noisy measurements is one of the essential requirements in numerous real time scientific and engineering problems. Therefore, accuracy is the primary constraint in applications such as target tracking, automotive land vehicle and flight control systems, non-linear process control and optimization, real time surveillance and life safety applications etc. [1,2]. However, in most of every day and real-life applications, measurement model depends upon the characteristics of receiving data, such as Global Satellite Navigation System (GNSS) signals, inertial sensors (accelerometer, gyroscope), ranging and visual sensors, nonlinear power management systems etc. Calibration errors, measuring system failure and measurement accuracy limits are some of the main causes that can bias the source system observations. In applications, those depend upon the range sensors [3], contamination within measurements grows as the relative distance between the measuring system and object increases, e.g., infrared (IR) distance measurement sensors. In other systems, e.g., vision sensor measurements [4], the amount of contamination grows with the size of data. In systems such as image processing [5], control systems [6], communication systems [7] and tracking systems [8], observations are contaminated with multiplicative noise. Therefore, research towards the development of algorithms for precise and reliable state estimation of nonlinear systems has received considerable attention. The primary objective of a decent estimation algorithm is to provide reliable system dynamics in the presence of noisy observations.

The presence of outliers severely degrades the reliability of the state estimation process of non-linear dynamic systems. In most of state-of-the-art estimation techniques (e.g., Kalman filters, particle filters, nonlinear least square estimation, etc.), observation non-linearities are not treated discretely and assumed to be corrected along with modeled noise during estimation process. However, outlier observation’s impact on the estimates is more severe and cannot be treated the same as model errors. In addition, the accuracy of estimated states also directly depends upon the error models of system dynamics which are established based on predefined assumptions. The accuracy of the estimation process is highly impaired in the case of divergence of the defined assumptions. In the case where system dynamics diverge from assumptions, most likely the estimates will diverge or at least will not be as useful. Hence, for reliable state estimations, the process must likely be able to handle the divergence from model assumptions. 

## 2. Background and Literature Review 

There are several types of error sources which effects the estimation accuracy, specifically in filtering-based techniques. Outliers, as pointed out by Gandhi in [9], can be classified into innovation, structural and measurement outliers, as shown in the Table 1.

From the defined outlier types, observation outliers originate from the measuring systems themselves such as random walk, Gaussian distributed errors, measurement error drifts, etc. or can be caused by a poor system calibration process. Observation outliers’ results in wrong measurements that can lead to divergence of the system from the true state or to wrong convergence points. In contrast, innovation outliers are caused by poor system state dynamic assumptions, i.e., a stable system assumption for an unstable system, wrong state forward and process noise models. Innovation outliers affect the accuracy of system state prediction and the process noise transfer model. Structural outliers are caused by wrong system models as a whole, for example a system structure where states are wrongly modeled against true system dynamics and/or true observations. As a result, observation and innovation outliers can be handled through robust state estimation processes, however, it is not possible to remove or recover from structural outliers.

Multiple estimation models, including filter-based and direct estimation procedures, have been proposed in the literature to minimize the effect of outlying observations on final estimates. In direct estimation approaches, the present state depends only on the current measurement and not affected by prior measurements or states. These approaches are specifically useful in situations where estimation/measurement errors do not keep on in future predictions such as GNSS-based localization systems, data analysis frameworks in statistics and econometrics, etc. Least squares estimation (LSE) and its variants [10], the Gauss–Newton (GN) estimation algorithm and the maximum likelihood estimator (MLE) and its variants, etc. are some of the sophisticated examples of direct estimation techniques. In filtering-based approaches, system states are updated in a timely way using current observations and prior estimates. Therefore, the prior estimated state is as important as measurements. In comparison to observation-only dependent techniques, filtering-based approaches are more useful in scenarios where the system states are continuously tracked over time depending on prior estimates. 

The Kalman filter (KF) is one of state-of-the-art estimation algorithms that has been extensively studied for all kinds of state estimation problems for noisy measurements in discrete-time dynamic systems. The primary objective of the KF is to track the dynamic changing states in the presence of noisy and incomplete measurements. Classical KF statistically provides an optimal state of a linear system by incorporating various noise characteristics, in addition of a forward model and an observation model. However, KF is considered as the optimal filter only for linear systems having Gaussian distributed noise. On the other hand, most real world problems are non-linear, and the performance of the KF severally degrades when the assumptions related to system linearity and Gaussian distribution are violated. In order to enhance the performance and to overcome limitations of KF against system nonlinearities, Extended Kalman Filter (EKF) [11] was introduced. EKF handles the system nonlinearities through conversion of non-linear system equations into linear ones by applying a first order Tayler series approximation around the current mean error and covariance. Consequentially, it arrives at the best state estimate more quickly than conventional KF. However, first order Tayler approximation errors grow rapidly in the presence of strong nonlinearities within the function model. In order to circumvent these errors, two variants of EKF, the Unscented Kalman Filter (UKF) [12], and iterated extended Kalman Filter (IEKF) [13] are proposed as alternatives. IEKF iteratively linearizes the non-linear system functions for compensation of linearization errors, whereas the Unscented KF (UKF) controls the unscented linearization by deliberately giving sigma points for estimation of mean and error covariance of a random state vector resulting in a more accurate state than the EKF [14,15]. The UKF filter has been explored by many researchers as the solution of nonlinearity functions in an extensive range of application domains [16,17,18]. Either centralized [19] or decentralized [20] UKF structures, can significantly improve the computational efficiency. On the other hand, in [21] it has been proved that there is a lack of robustness that degrades the performance of UKF and/or EKF significantly in the presence of measurement outliers. In order to diminish this problem, in [20], a scheme based upon the normalized innovation vector is proposed for identification of observation outliers, despite of the fact the scheme is vulnerable to innovation errors. Another outlier detection scheme, based on Least Absolute Value (LAV) estimation, is proposed in [22]. Despite of the fact that the proposed schemes can handle observation outliers, they do not cover the state prediction outliers caused by calculations in the state approximation model and/or because of system process noise. In some other variants of conventional KF, researchers adopted diverse statistical tools in order to overcome the KF limitations. In [23], authors proposed that adaptation of maximum correntropy criterion (MCC) can enhance the KF performance under non-Gaussian noise conditions. In [24], the author provides the generalization of correntropy as MCC and its applications for non-Gaussian signal processing. However, the results indicated that correntropy ultimately depends upon the kernel size that must be selected in accordance to the application constraints and additional measures are required for kernel size selection. In [25], wavelet transform (WT) is used to improve the efficiency of a framework with two parallel adaptive unscented Kalman filters (AUKFs) for estimation of state of charge (SOC) of a LiFePO_4_ battery. However, the proposed framework is only beneficial in applications where the system model precisely (or closely) defines the system characteristics having very little rate of change. In [26], authors proposed a wavelet filter banks-based scheme for handling singularities and applied it in a watermarking scheme.

In this paper we have developed a framework for handling system and measuring model nonlinearities either because of modeled or unmodeled errors. In our prior work [27], we studied and proposed a robust estimation algorithm for accuracy enhancement founded on statistical estimation models. The proposed scheme is only dependent upon current observations. The primary objective of the current work is to maintain consistency and continuity in system state tracking at higher accuracy. The proposed framework is founded on two schemes: the KF approach and the work proposed in [27]. Our main contributions in this work are:

(1) A filtering-based optimization scheme founded on IEKF process.
(2) Robust state approximation and error covariance estimation model.
(3) Robust dynamic state update model built on a robust statistical estimation process [27].

The proposed estimation model is named Robust-Reweighted IEKF. The rest of the paper is organized as follows: in Section 3, conventional EKF, its limitations and IEKF are revisited. In Section 4, the proposed estimation model is derived and discussed in detail. In Section 5, the performance of the proposed scheme is analyzed and discussed for numerical simulation and real data. The current and the future work is concluded in the last section.

## 3. Extended Kalman Filter Modeling

The extended Kalman Filter (EKF) is one of the most widespread tools for state approximation or data assimilation for non-linear dynamic systems. In EKF, the state estimation problem is carried out sequentially, where first the model states are approximated using prior system states and are then updated from the new available observations.

Every time a set of measurements $y(t_{k})$ is available, the filter uses the observations to update a priori approximated states at time instance $t_{k}$, denoted as $\overset{⌢}{x}\left(t_{k},t_{k-1}\right)$, to update system states $\overset{⌢}{x}\left(t_{k}\right)$, along the transformation of the a priori process error covariance matrix $\overset{⌢}{\Sigma }\left(t_{k},t_{k-1}\right)$ to a posteriori process error covariance estimate $\overset{⌢}{\Sigma }\left(t_{k}\right)$. Updated state estimates are propagated to the next time instance $t_{k+1}$ for a priori state prediction. Because the system dynamics are non-linear, the state propagation is done by integration of the following dynamic equation:

(1) $$\frac{d}{dt}\overset{⌢}{x}=f\left(x,t,w\right)\Rightarrow \dot{x}=f\left(x,t\right)$$

Within the above equation, $x\left(t_{k}\right)=x_{0}$ is the initial condition, and f is a nonlinear function in x and w is the process noise and assumed to be Gaussian with zero mean and with covariance $Q\Rightarrow \left[p\left(w_{k}\right)∼N\left(0,Q_{k}\right)\right]$. Similarly, the measurement model relates x(t) to y(t) with the function of h(x,t,v)⇒h(x,t), with zero mean measurement noise vector v with the error covariance of $R\Rightarrow \left[p\left(v_{k}\right)∼N\left(0,R_{k}\right)\right]$. The sequential procedure of the EKF is summarized in the following two steps.

### 3.1. State Approximation Model

With an initial state $\overset{⌢}{x}\left(t_{k-1}\right)$ with covariance $\overset{⌢}{\Sigma }\left(t_{k-1}\right)$ and observations $y\left(t_{k}\right)$:

(1) First step involves state transition matrix and system states approximations between two time instances from $t_{k-1}$ to $t_{k}$. Neglecting higher order terms, the linearized system model function can be expressed as:
  (2) $$\overset{⌢}{x}\left(t_{k},t_{k-1}\right)=A\left(t_{k}\right)\overset{⌢}{x}\left(t_{k}\right)$$
  In the above expression, A is a *m* × *m* continuous time state transition matrix based on partial derivatives of f with respect to $\overset{⌢}{x}$, $\left[A\left(t\right)=\partial f/\partial \overset{⌢}{x}\right]$ and a discrete state transition matrix Φ is obtained by assimilation of the matrix expression below:
  (3) $$\dot{\Phi }=\frac{d}{dt}\Phi \left(t_{k},t_{k-1}\right)=A\left(t_{k}\right)\Phi \left(t_{k},t_{k-1}\right)$$
  As the initial expression Φ is considered as a *m* × *m* identity matrix, the solution of state transition equation is expressed as:
  (4) $$\overset{⌢}{x}\left(t_{k},t_{k-1}\right)=\Phi \overset{⌢}{x}\left(t_{k-1}\right)$$
(2) In a similar manner, the forward model error covariance is projected to the next time instance using the following expression:
  (5) $$\overset{⌢}{\Sigma }\left(t_{k},t_{k-1}\right)=\Phi \left(t_{k}\right)\overset{⌢}{\Sigma }\left(t_{k-1}\right)\Phi {\left(t_{k}\right)}^{T}+Q$$

### 3.2. Observation Update Model

(1) The observation update operator involves the calculation of the observation residual vector $\Delta y\left(t_{k}\right)=y\left(t_{k}\right)-h\left(\overset{⌢}{x}\left(t_{k},t_{k-1}\right)\right)$ and observation Jacobian matrix based on partial derivatives of observation vector w.r.t $\overset{⌢}{x}\left(t_{k},t_{k-1}\right)$, $\left[H=\partial h/\partial \overset{⌢}{x}\right]$.
(2) Computation of the Kalman gain matrix using the following expression:
  (6) $$K_{k}=\overset{⌢}{\Sigma }\left(t_{k},t_{k-1}\right)H_{k}^{T}/\left(H_{k}\overset{⌢}{\Sigma }\left(t_{k},t_{k-1}\right)H_{k}^{T}+R\right)$$
(3) Update the estimated states using the current observations:
  (7) $$\overset{⌢}{x}\left(t_{k}\right)=\overset{⌢}{x}\left(t_{k},t_{k-1}\right)+K_{k}\left(y\left(t_{k}\right)-h\left(\overset{⌢}{x}\left(t_{k},t_{k-1}\right)\right)\right)$$
(4) Update the forward model error covariance matrix:
  (8) $$\overset{⌢}{\Sigma }\left(t_{k}\right)=\left(I_{m\times m}-K_{k}H_{k}\right)\overset{⌢}{\Sigma }\left(t_{k},t_{k-1}\right)$$

Every time a new observation is available, EKF provides a new state estimate. For a real time solution, corrections are added to the system states for each observation. Moreover, partial derivatives of system dynamic functions are recomputed along with each observation resulting in a more accurate updated state transition matrix.

### 3.3. Limitations of EKF

EKF is seriously capable of tracking non-linear system dynamics with higher accuracy. However, the performance of EKF is degraded because of truncation, system modeling, linearization and cumulative rounding errors, in addition to unknown non-Gaussian noise. The impact of these errors can be reduced to some extent by taking a few careful measures such as increasing the observation frequency, a careful filter calibration process and offline data tests to design an appropriate model for the system dynamics. Despite all efforts, the performance of EKF is badly influenced by an increasing amount of nonlinearities and the wrong state transition model that results in divergence from true system dynamics.

### 3.4. Iterative Extended Kalman Filter

An alternative solution to overcome the EKF limitations is the IEKF estimation model. In IEKF, linearization is achieved in multiple iterations based on an initial guess $\overset{⌢}{x}\left(t_{k},t_{k-1}\right)$. Within each iteration, $h_{k}$ and $H_{k}$ are evaluated around the new reference state. The observation update operator from EKF, is replaced with the following equation during the iterative process:

(9) $$\bar{x}\left(t_{k}\right)=\overset{⌢}{x}\left(t_{k},t_{k-1}\right)+K_{k}\left(y\left(t_{k}\right)-h\bar{x}\left(t_{k}\right)+H_{k}\left[\overset{⌢}{x}\left(t_{k},t_{k-1}\right)-\bar{x}\left(t_{k}\right)\right]\right)$$

In the above expression, $\bar{x}\left(t_{k}\right)$ represents a temporary updated state during each iteration. Once $\bar{x}\left(t_{k}\right)$ attains an optimal state, either by threshold or by number of iterations, the final state is the optimal estimate of the system model. In other words, IEKF is analogous to an optimization technique to attain an optimal state.

The complete IEKF process is summarized in Algorithm 1.

**Algorithm 1.** IEKF estimation model
(1)***Step 1 (initialization)***: Initialization of IEKF is similar to EKF as the initial state vector ${\tilde{x}}_{0|0}$ and initial error covariance ${\bar{P}}_{0|0}$, and state prediction step are the same as in EKF.(2)***Step 2 (measurement update iterations)***: Measurement iterations are started by initializing ${\hat{x}}_{t+1}^{0}=x_{t+1|t}$ and i=0, computation of Jacobian matrix, Kalman gain and state estimate for the next iteration:$$H_{t+1}^{i}={\frac{\partial h_{t+1}\left(s\right)}{\partial s}|}_{s={\hat{x}}_{t+1}^{i}}$$
$$K_{t+1}^{i}={\bar{P}}_{t+1|t}{\left[H_{t+1}^{i}\right]}^{T}{\left(H_{t+1}^{i}{\bar{P}}_{t+1|t}{\left[H_{t+1}^{i}\right]}^{T}+R_{t+1}\right)}^{-1}$$
$${\hat{x}}_{t+1}^{i+1}={\hat{x}}_{t+1|t}+K_{t+1}^{i}\left(z_{t+1}-h_{t+1}\left({\hat{x}}_{t+1}^{i}\right)-H_{t+1}^{i}\left({\hat{x}}_{t+1|t}-{\hat{x}}_{t+1}^{i}\right)\right)$$Step 2 is iteratively executed once a stopping criterion is achieved, i.e., the difference between two successive approximations is less than a predefined threshold ξ, $\left\|x_{t+1}^{i+1}-x_{t+1}^{i}\right\|<\xi$.(3)***Step 3 (finalization)***: Once the stopping criteria is achieved, the state vector and covariance matrix is finalized


**Figure 1:**
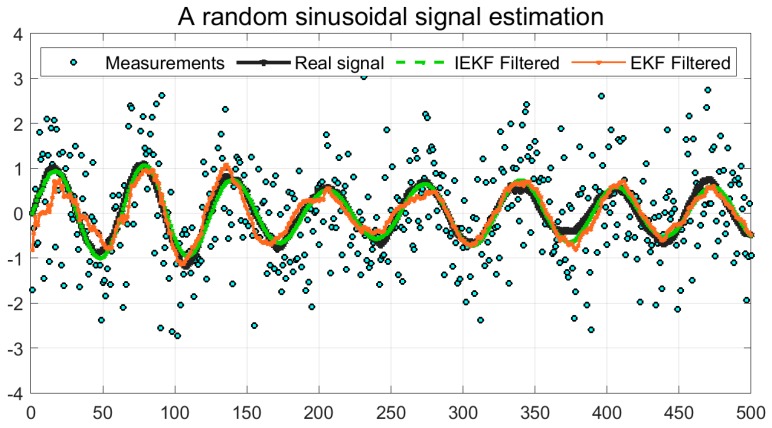
Comparison of EKF and IEKF over a sinusoidal signal.

Figure 1 presents a simple comparison of EKF and IEFK over a sinusoidal wave with a small amount of additive White Gaussian noise. The estimation error in terms of root mean square error is shown in the Table 2.

## 4. Robust Reweighted Iterative Kalman Filter

IEKF can only attain the optimal states in the presence of an error-free state prediction model, and having a limited number of observation outliers. Although, IEKF can attain an optimal state in the presence of observation outliers, it has minimal effect on state innovation outliers (errors). In order to manage the non-linearity within the system model and outliers within observations, in this section, a robust reweighted iterative extended Kalman filter (RRIEKF) is proposed. Before derivation of the proposed filtering algorithm, some details related to problem formulation and robust estimation are discussed.

### 4.1. State Estimation Robustness

An optimal estimator is focused on predicting state variables maintaining the efficiency and robustness. By robustness it means that the estimator must preserve a small variance within observations though observations are not following the prior (initial) system model assumptions related to innovation and measurement errors. Theoretically, a robust estimator defines an estimation model that fits most non-linear systems. In the robust estimation process, the influence of the outlying observations is minimized by reducing their weights throughout an estimation process. Additionally, robust estimation models can also be used to detect and remove observation outliers within highly non-linear and dynamically changing system models. Let us consider an estimation function T having an observation Z with *n* number of total data points and *m* number of contaminated observations where $T(Z)=\bar{\beta }$. The effect of contaminated observations on the true estimate and the breakdown point (BDP) of the estimation function can be represented as:

(10) $$\begin{array}{l} effect\left(m;T,Z\right)=\underset{\vec{Z}}{\sup}\left\|T\left(\vec{Z}\right)-T\left(Z\right)\right\|\\ BDP\left(T,Z\right)=\min\left\{\frac{m}{n}:effect\left(m;T,Z\right)\right\} \end{array}$$

BDP provides a measure of contaminated observations for estimator failure. On the contrary, the relative efficiency of an estimation function can be defined as the ratio of the real variance to the possible minimum variance [28]. Relative efficiency provides the comparison among the efficiency of any estimator to some popular estimation techniques. Mathematically, the relative efficiency of two estimation functions, $T_{1}$ and $T_{2}$ can be described as:

(11) $$efficiency\left(T_{1},T_{2}\right)=\frac{E\left[{\left(T_{1}-\bar{\beta }\right)}^{2}\right]}{E\left[{\left(T_{2}-\bar{\beta }\right)}^{2}\right]}$$

The influence function is another robustness parameter that describes the effect of very small contamination bias at some random point. Mathematically, the asymptotic influence function for an estimation algorithm Є using first derivative with minimal distribution ${Ÿ}_{Є}$ is given by:

(12) $$IF(x,Є,{Ÿ}_{Є})=\underset{\iota \to 0}{\lim}\frac{{Є}_{\infty }\left(Ÿ\right)-{Є}_{\infty }\left(F_{Є}\right)}{\mu }={\left[\frac{\partial {Є}_{\infty }\left(Ÿ\right)}{\partial \mu }\right]}_{\iota \to 0}$$

Robustness properties, i.e., boundedness and continuity, are satisfied by the influence function. The former property ensures that the presence of the small segment of bias or outlier should have a small effect on the estimate. The latter property guarantees a little change in the guess in the presence of a small change in data. A filter satisfying both properties has robustness in terms of qualitative robustness.

### 4.2. Robust State Prediction Model

The proposed estimator is developed on four key foundations of filtering-based estimation approaches, first the final estimate is highly dependent upon the initial guess. A reliable and bounded initial guess can quickly lead to a convergence point. For a reliable initial guess, a window of *n* former estimates is maintained and an initial prediction is optimized using current and prior estimates. The second step involves the pre-whitening of observations through robust statistics in order to uncorrelate the predicted and measured observations. The pre-whitening process involves the batch mode prediction model. Thirdly, a robust process error covariance matrix is predicted prior to starting the iterative optimization process. Finally, robust regression statistics are involved in an iterative manner to minimize the error residual between estimated and measured observations.

#### 4.2.1. Batch Mode State Prediction Model

For a reliable state prediction, a window is maintained over *n* prior estimates along time. Prior states are processed in batch mode regression simultaneously for maintaining data redundancy. Data redundancy improves the capabilities of the estimator for dynamic state estimation and helps to suppress outliers. Let us consider a non-linear dynamic system with some prior information related to system dynamics and prior distribution $\hat{ø}∼N\left(ø,{Л}^{-1}\right)$ that translates to a single point state estimate. The prior distributed information is fragmented into four components as follows:

(13) $$\hat{ø}∼N\left(\left[\begin{array}{l} {ø}_{k}\\ {ø}_{m,1:n} \end{array}\right],\left[\begin{array}{cc} {Л}_{k} & {Л}_{km}\\ {Л}_{km}^{T} & {Л}_{m} \end{array}\right]{Л}^{-1}\right)$$

The probability density function (PDF) of prior information is expressed as;

(14) $$þ\left(\hat{ø}\right)={ή}_{\hat{ø}}\exp\left\{\frac{-1}{2}{\left(\hat{ø}-ø\right)}^{T}Л\left(\hat{ø}-ø\right)\right\}$$

In the above expression, ${ή}_{\hat{ø}}=\sqrt{{\left(2\pi \right)}^{\left|\hat{ø}\right|}\det\left({Л}^{-1}\right)}$ is the normalizing feature. Thus the posterior probability of the dynamic state estimate can be expressed as:

(15) $$þ\left(ø|y\right)=þ\left(y|\hat{ø}\right)þ\left({ø}_{p}\right)þ\left(\hat{ø}\right)$$

Our objective is to compute an aposteriori estimate that results in maximum density function. The required objective can be attained by grouping the dynamic system model, stated forward process model and prior information in batch-processing regression form as follows:

(16) $$\gamma \left(ø\right)=\left[\begin{array}{l} \gamma_{f}\left(ø\right)\\ \gamma_{\pi }\left(ø\right) \end{array}\right]=\left[\begin{array}{l} {ø}_{p}-f\left(ø\right)\\ \hat{ø}-\left[\begin{array}{l} {\hat{ø}}_{p1}\\ {\hat{ø}}_{m} \end{array}\right] \end{array}\right]$$

(17) $${Ū}^{-1}=\left[\begin{array}{ccc} R^{-1} & 0 & 0\\ 0 & Q^{-1} & 0\\ 0 & 0 & Л \end{array}\right]$$

The above expression defines the error covariance matrix for the initial estimate.

#### 4.2.2. Robust Covariance Estimation

It is well known that the performance of the Kalman filter is highly dependent upon the exact knowledge of system dynamics and statistical noise properties such as process noise $Q_{k}$, and measurement noise $R_{k}$. However, because of experimental errors, system model errors, etc., it is likely to be impossible to satisfy the ideal assumptions. Therefore, to improve the performance of the proposed estimation algorithm, a robust error covariance estimation process is introduced in a recursive manner. Statistically, the covariance of two random variables can be obtained as:

(18) Cov(x,y)=E(x,y)−E(x)E(y)

Considering a random variable ζ=w+v, where w and v are process and measurement noise respectively, and the covariance can be estimated as follows:

(19) $$Cov\left(\zeta \right)=E\left(\zeta \zeta^{T}\right)=\underset{n\to \infty }{\lim}Cov\left(\zeta \right)=\underset{n\to \infty }{\lim}\frac{1}{n}\sum_{i=1}^{n}\zeta_{i}\zeta_{i}^{T}$$

(20) $$Cov\left(\zeta \right)=\underset{n\to \infty }{\lim}\frac{1}{n}\sum_{i=1}^{n}\zeta_{i}\zeta_{i}^{T}-R$$

Considering $Cov_{n-1}\left(\zeta \right)$ as the covariance estimated from the observation to n−1 time instance, the current covariance can be estimated using following expression:

(21) $$\begin{array}{ccc} Cov_{n}\left(\zeta \right) & = & \frac{1}{n}\sum_{i=1}^{n}\zeta \zeta^{T}\\ & = & \frac{1}{n}\left(\sum_{i=1}^{n-1}\zeta \zeta^{T}+\zeta \zeta^{T}\right)\\ & = & \frac{1}{n}\left(Cov_{n-1}\left(\zeta \right)+{\left(\zeta \zeta^{T}\right)}_{n}\right) \end{array}$$

Using the definition that $Cov_{n}\left(\zeta \right)-Co\underline{v}\left(\zeta \right)\approx 0$ as n→∞, a robust error covariance is produced using current and prior n−1 observations. Therefore, as a new observation is available at time instance *n,* new error covariance is computed using the algorithm as described in the following.

Having initial estimate ${\tilde{x}}_{0|0}$, initial covariance ${\bar{P}}_{0|0}$, state transition matrix Φ, and by making the assumption that Cov(w)=Q and Cov(v)=R, observations can be modeled through the following expressions. The relation between observation and states is represented as:

(22) $$y_{k}=Hx_{k}+v_{k}$$

Using Equation (4), Equation (22) is modified as:

(23) $$y_{k}=H\left(\Phi x_{k-1}+Gw_{k-1}\right)+v_{k}$$

According to the least squares estimation (LSE) principle:

(24) $$x_{k-1}={\left(H^{T}H\right)}^{-1}H^{T}y_{k-1}$$

Replacing Equation (24) in Equation (23):

(25) $$y_{k}=H\left(\Phi {\left(H^{T}H\right)}^{-1}H^{T}y_{k-1}+Gw_{k-1}\right)+v_{k}$$

and by replacing $B={\left(H^{T}H\right)}^{-1}H^{T}$, Equation (24) can expressed as:

(26) $$\begin{array}{ccc} y_{k} & = & H\left(\Phi By_{k-1}+Gw_{k-1}\right)+v_{k}\\ & = & H\Phi By_{k-1}+HGw_{k-1}+v_{k} \end{array}$$

According to the definition that ${\left(H^{T}H\right)}^{-1}H^{T}H=I$ and by multiplying B on both sides of Equation (26):

(27) $$\begin{array}{l} By_{k}=\Phi By_{k-1}+Gw_{k-1}+Bv_{k}\\ By_{k}-\Phi By_{k-1}=Gw_{k-1}+Bv_{k} \end{array}$$

Representing $\zeta =By_{k}-\Phi By_{k-1}$, the state error covariance can be computed in recursive manner. Using Equation (21) and maintaining a window $\left[\zeta_{1}\;\zeta_{2}\;\cdots \;\zeta_{n}\right]$ over time:

(28) $$Cov_{k}\left(w\right)=Cov_{k}\left(\zeta \right)-R$$

### 4.3. Iterative Transfer Model for System State Optimization

The primary objective of robust filtering is to minimize the residual function. The required objective is achieved through projection statistics within the robust filtering process in an iterative manner. According to the standard definition that using projection statistics in estimation process, the estimator can track possible future changes in observations only if the assumptions about the system dynamics are actually occurring. The projection statistics of the ith row value, ${đ}_{i}$, for the jth state predictions are represented in the following expression:

(29) $$P_{i}^{s}=\underset{\left\|u\right\|=1}{\max}\frac{\left|{đ}_{i}^{t}u-\underset{j}{med}\left({đ}_{j}^{t}u\right)\right|}{1.4826\underset{i}{med}\left|{đ}_{i}^{t}u-\underset{j}{med}\left({đ}_{j}^{t}u\right)\right|}$$

$P_{i}^{s}$ statistics provides the outliers information when compared against a defined threshold. For a system having *n* observations, the optimal state estimation algorithm is described in the following sections.

#### 4.3.1. Subsampling Optimization

A subsampling process is involved just in case enough observations are available against the minimum requirement. The primary objective of the subsampling optimization is to increase the redundancy of error-free solutions. In the subsampling process, all candidate solutions are optimized iteratively. However, if it is necessary, all observations are considered as a single subsample. Using the least square principle:

(30) $${\vec{\gamma }}_{p}={\left({\vec{H}}_{p}^{T}W{\vec{H}}_{p}\right)}^{-1}W{\vec{H}}_{p}^{T}y_{p}$$

In Equation (30), ${\vec{\gamma }}_{p}$ represents the solution of $p^{th}$ subsample. $\vec{H}$ represents the Jacobian for observations used to compute ${\vec{\gamma }}_{p}$. W is the weight matrix that is unknown at start and is initialized as an identity matrix. A standardized residual, ${\ddot{\gamma }}_{p}=\left(y_{p}-{\vec{H}}_{p}{\vec{\gamma }}_{p}\right)/s_{p}^{o}$ is computed, where $s_{p}^{o}$ is the scale factor and is computed as follows:

(31) $$s_{p}^{o}=\left\{ \begin{array}{ll} \frac{median\left|{\vec{\gamma }}_{p}-median\left({\vec{\gamma }}_{p}\right)\right|}{0.6745}, & \mathrm{iteration}=1\\ \sqrt{\frac{1}{nK}\sum_{i=1}^{n}w_{i}{\vec{\gamma }}_{p}^{2}}, & \mathrm{iteration}>1 \end{array}\right.$$

Using a standardized residual vector, observation weights are calculated according to the following expression:

(32) w⇀i(γ¨p)={{[1−(γ¨pi1.547)2]2,|γ¨pi|≤1.5470,|γ¨pi|>1.547,itreation=1ρ⇀(γ¨pi)γ¨p2i,      iteration>1

Weights are applied in Equation (30) to update the estimate.

#### 4.3.2. Iterative Extended Kalman Filter

Once the system states are updated using weighted least squares (WLS), the updated states are used in the IEKF observation model using Equation (9) in an iterative manner to determine the optimal point. If more than one solution are available, Equation (9) is applied to all solutions from the first step. The residual vector is computed between recent and last estimates and is used to compute a scale factor. Using Equation (31), weights are computed using Tukey’s biweight function as:

(33) w⇀j={[1−(γ¨j4.685)2]2,|γ¨j|≤4.6850,|γ¨j|>4.685

In the above expression, 4.685 is selected as the threshold as it provides a 50% break down point (BDP). Table A1 is shown in Appendix A. The iteration process is stopped once the single and/or multiple solutions attain the optimization conditions:

(34) ‖xpi−xpi−1‖‖xpi‖<kcc

In the above expression, $k_{cc}$ is the optimization and threshold with the value of 0.001. Otherwise, the complete process is iterated from Equation (30) either for a single or multiple solutions.

In the case of a single solution, the optimized state in an iterative process is taken as the final state. On the contrary, for multiple solutions, an optimized state having a minimal scale factor is considered as final state:

(35) $${\overset{⌢}{x}}_{k}\left(t_{k}\right)=x_{j}\;\left(x_{j}\in s_{j}^{o}∍s_{j}^{o}<s_{i}^{o}\;\forall i=1,2,\cdots ,p\right)$$

(36) $$\Sigma \left(t_{k}\right)=\left(I_{m\times m}-K_{k}H_{k}\right)\Sigma \left(t_{k},t_{k-1}\right)$$

Here $K_{k}$ and $H_{k}$ are the Kalman gain and observation Jacobian computed from the optimal state estimate.

## 5. Discussions and Numerical Analysis

In this section, some key aspects of the proposed estimation algorithm are discussed. Later on, the performance of the proposed algorithm is analyzed. Primarily, all filtering-based approaches consists of two key steps, state prediction, and state approximation. State prediction steps involve an initial guess of future changes in system dynamics. This initial guess is made by considering the last approximation as a true system state and using a system dynamics model. Later on, the initial guess is corrected using system observations. However, if either the initial guess or correction model deviate from the true dynamics, this error will pass on in future estimates and may result in a complete system failure. In our proposed estimation procedure, inconsistency in the initial guess is handled through batch mode processing. The key objective of using a batch mode model is to attain reliability and consistency at the state prediction level through increasing redundancy. Another important aspect to enhance the estimation accuracy is the precise knowledge of the process error covariance matrix. Here, we provided an online scheme for handling the process error covariance over time adaptively. The second phase of the algorithm involves the correction of the initial guess using observations. To reduce the influence of noisy/outlying observations, a weighting model is adopted for calculating and assigning weights to measurements. This process is iteratively performed to achieve an optimal estimate. Within every iteration, new weights are calculated and the prior estimate is updated. A subsampling process is used in numerous statistical estimation techniques to provide a generic understanding of the data in robust and multivariate regression theory [29,30]. The subsampling concept is adopted as an optional process in our proposed algorithm to improve the precision of the final estimate by increasing the redundancy of error-free estimates and to choose a best estimate based upon some predefined conditions.

Most of the research focused towards robust filtering (specifically Kalman) is for model-based systems where the system dynamics are stable or are changing very slowly. Within these algorithms, the primary objective is to handle observation nonlinearities [18,19,20,23,25]. However, very little research is available in the literature for handling multirate sensor integrations. In [31], a six degree of freedom (DoF) estimation algorithm is provided using iterative closest point (ICP) and an adaptive Kalman filter (AKF) for laser scanner and IMU (inertial measurement unit) integration and compared against conventional KF. KF is used for IMU bias with the assumption that IMU noise is fixed and with known characteristics. Although this assumption is acceptable for highly accurate expensive mechanical IMUs, it is not applicable to low cost MEMS (micro-electro-mechanical systems) IMUs. In [32], an integration scheme for GNSS, ultra wideband (UWB) and IMU is proposed using a robust Kalman filter (RKF) and compared against conventional KF. However, the primary focus in this work is to prove the localization accuracy enhancement using integrated navigation in GNSS-denied environments and less on KF. A new approach, maximum correntropy unscented Kalman Filter (MCUKF) is proposed in [33] and compared against conventional filters. MCC is new approach that is effective for handling non-Gaussian type noise. However, MCC performance depends on the kernel size that needs to be selected. Besides good results in [33], the key issue is the lack of a kernel size bandwidth selection scheme that is an additional effort. In [34], a neural network (NN)-enhanced adaptive robust Kalman filter is proposed for GNSS/INS integration. The primary objective of using NN is to provide an online training scheme for selection of outlying GNSS observations and prior training is required. On the other hand, a key aspect of our proposed algorithm is its two-fold advantage. Firstly, it provides a generic algorithm for state prediction in complex model-based nonlinear systems. Secondly, it is highly effective for multirate multisensory integration. We proposed a scheme in [27] for detection and exclusion of outliers without any prior knowledge and used it in RRIEKF with some modifications. 

Performance of the proposed algorithm is assessed using extensive numerical simulations and real sensor measurements for multirate sensor data integration. Numerical simulations provide a way of performance assessment in a controlled environment under defined conditions such as number of outliers, scenario complexity, noise level, etc. On the contrary, physical measurements show the performance in real stable and dynamic environments. For performance assessment, conventional EKF, UKF and IEKF results are compared against the proposed estimator. For numerical analysis, datasets are simulated using non-linear state models under Gaussian distributed noise conditions. For true scenario analysis, inertial, and UWB sensor measurements are integrated over time for system localization. However, for creation of complex scenarios, additional noise and outliers are introduced within the sensor observations. 

### 5.1. Numerical Simulations 

**Figure 2:**
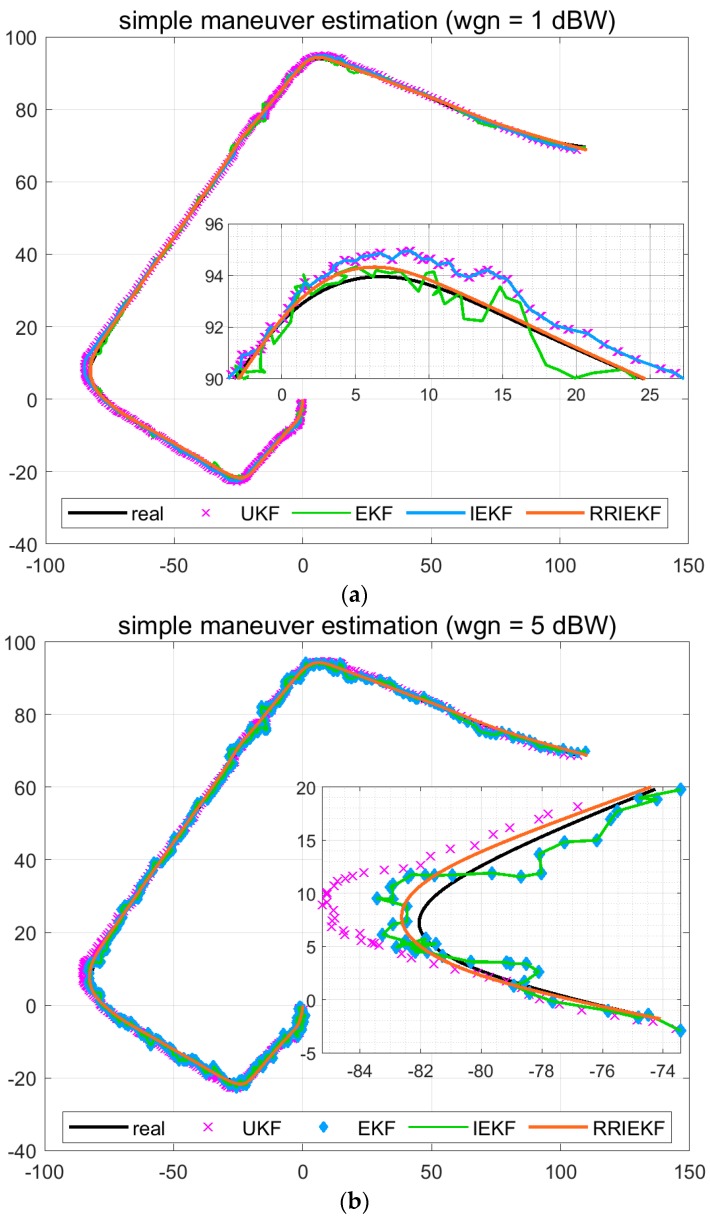
(**a**) Simple accelerative and turning movement estimation at 1 dBW WGN. (**b**) Simple accelerative and turning movement estimation at 5 dBW WGN.

In this subsection, two scenarios are simulated. The first scenario involves an accelerative and turning model maneuver with additive noise. The simulated trajectory and tracking results for UKF, EKF, IEKF and RRIEKF are shown in Figure 2a and in Figure 2b from measurements having 1 dBW and 5 dBW Gaussian distributed noise respectively. 

**Figure 3:**
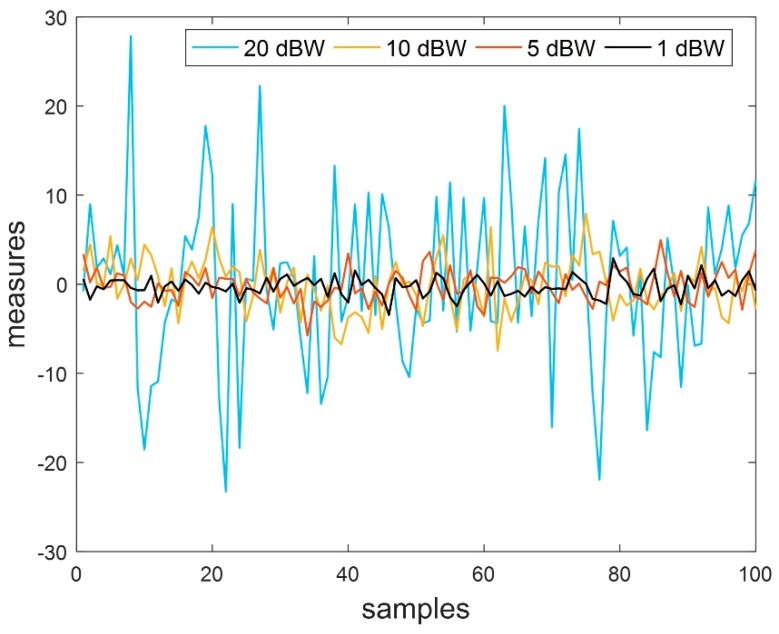
Noise-only measurements at different noise power levels.

The tracking performance over turn provides more realistic analysis of an estimation algorithm. The Figure 2a,b are expanded at two different turns in the maneuver for deep understanding of estimation behavior. In Figure 2a, UKF and IEKF tracked the object movement smoothly over turn, although have lack of accuracy. On the other hand, EKF tracking results have lack of accuracy and smoothness simultaneously. However, the proposed estimation algorithm showed better performance in terms of smoothness and accuracy. A more closer look reveals that the tracking performance of three algorithms, UKF, IEKF and RRIEKF, is very close to real path prior of turn. In Figure 2b, the performance of UKF and IEKF are badly influenced because of noise while there is a little effect on tracking performance of the proposed estimation algorithm. However, Figure 2 only provides a closer look for movement tracking. The performance of estimation algorithms is compared in terms of estimation errors and root mean square errors (RMSEs). In Figure 3, white noise-only measurements are shown those are used for observation errors. 

**Figure 4:**
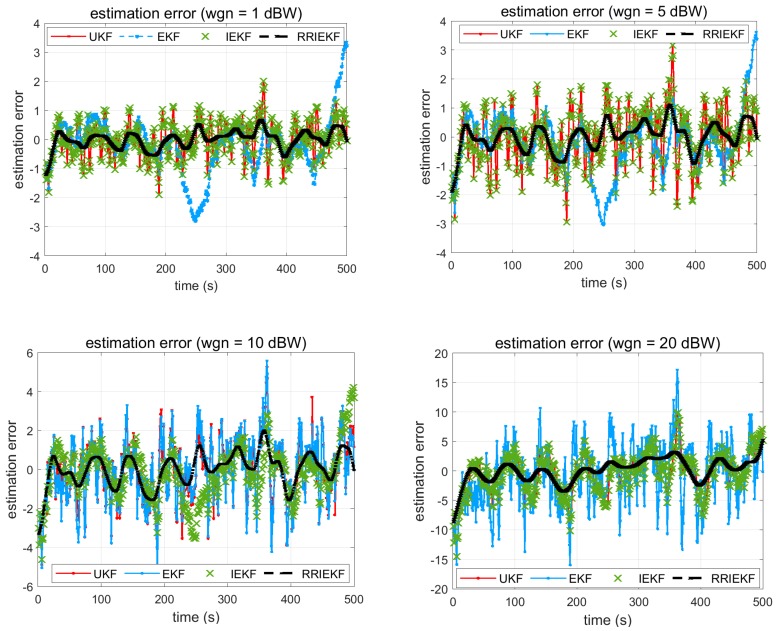
UKF, EKF, IEKF and RRIEKF estimation errors.

Estimation errors are shown in Figure 4. 

A careful analysis of Figure 4 reveals that the filters behaved differently under defined circumstances. The performances of UKF and IEKF are not much better than EKF in scenarios where the noise powers are 1 dBW and 5 dBW, respectively. Conversely, the performance of EKF is badly degraded once the noise power is increased to 10 dBW and/or 20 dBW. On the other hand, the proposed estimator, the RRIEKF filter, showed better performance at all noise levels, although the error is increasing as the noise power is changing from lower to higher, however, the same behavior is also indicated by other estimators. In Table 3, RMSE is compared. 

**Figure 5:**
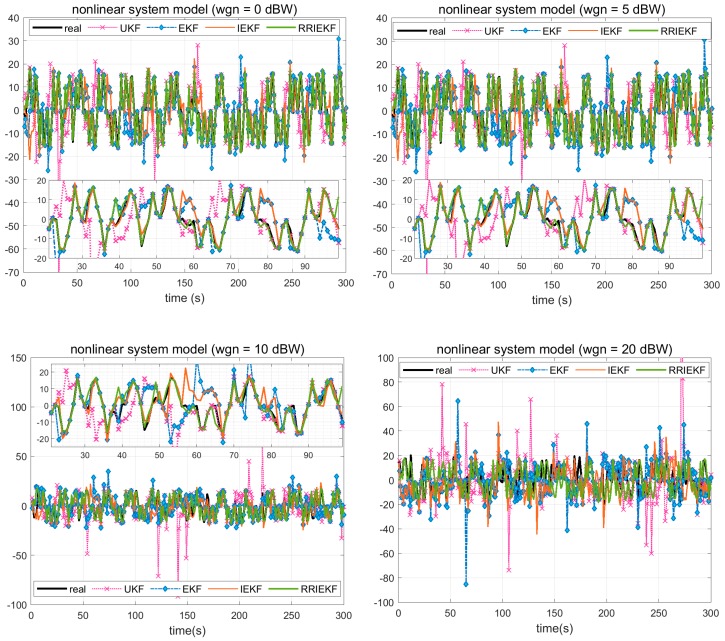
Performance over complexity.

For analysis of performance over complexity, a non-linear multivariate system is modeled with added Gaussian distributed noise. Estimation errors are shown in the Figure 5 and RMSE values are presented in Table 4. 

Estimation errors provide a generic overview of an estimation algorithm, however, step by step analysis provides a more realistic understanding. A deep analysis of Figure 5 and Table 4 reveals the superior performance of the proposed estimation algorithm in terms of RMSE in general and point to point state estimation for a complex nonlinear system.

**Figure 6:**
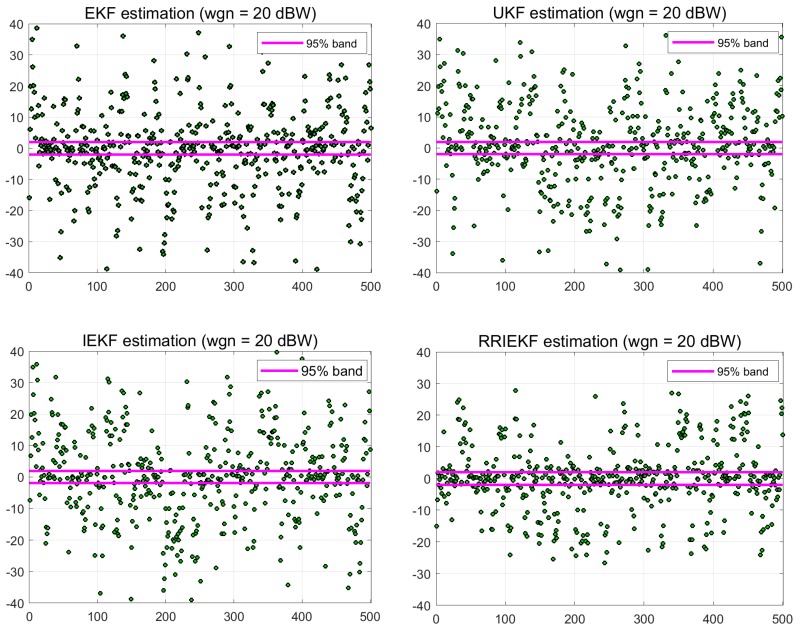
95% confidence level estimation residual index.

The performance of the UKF is a little better among the three filters, UKF, EKF and IEKF. However the performance of the proposed estimation process is far better than UKF. Another measure of the performance is the residual confidence level. In Figure 6, error residuals are presented in a 95% confidence level index. Residuals are compared at the maximum noise level of 20 dBW. The performance of the proposed estimation algorithm is better in two ways, first the error residual range is less than that of the other estimators, and also the index of residuals within 95% confidence interval is more than that of the other estimation filters. 

### 5.2. Real Data Analysis

The primary objective of this analysis is to prove the effectiveness of the proposed estimation algorithm for integration of noisy multirate sensors. Observations from IMU and UWB sensors are integrated over time for localization of moving bodies. The frequencies of the IMU and UWB outputs are 100 Hz and 25 Hz, respectively. IMU measurements are used in the state prediction model while UWB measurements are used for correction of the predicted states. Hence, compared to the observation frequency, the state prediction phase is very important because several times only noisy IMU measurements are used for localization. Therefore, it is an essential aspect to control errors at the state prediction level and this is handled through an online process error covariance estimation model. The results are compared in terms of estimated position errors and estimated speed. Table 5 presents the observation characteristics used in the estimation process.

**Figure 7:**
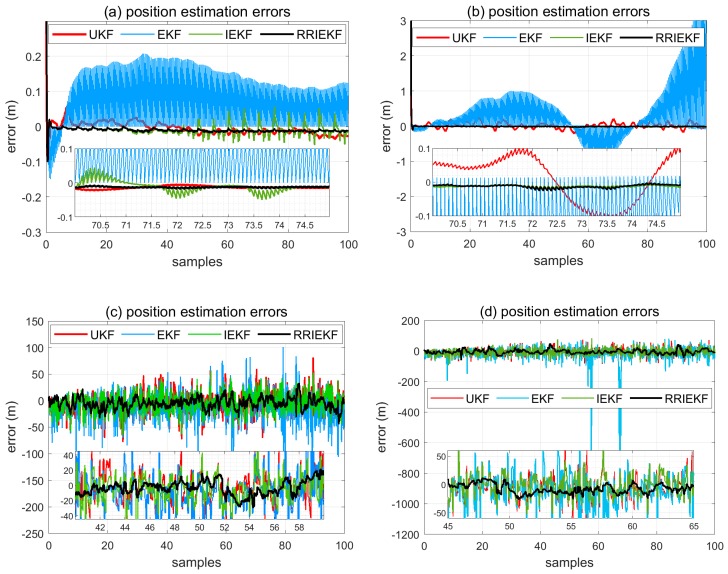
Position estimation error.

Position estimation errors from different filters are shown in the Figure 7 under different circumstances. Observations from low cost IMU and UWB sensors are very noisy. RMSE of estimated positions is shown in Table 6. 

In Figure 7a, observations are carefully calibrated for noise removal, and this is shown by the results that the estimation accuracy of all filters is appreciable. In Figure 7b, calibrated IMU observations are corrupted by adding noise at 20 dBW. In this case, the performance of the IEKF and RRIEKF is very close to being true. On the other hand, in Figure 7c,d, UWB sensor observations are corrupted with the AWGN noise source at 20 dBW level with one observation outlier in the latter case and the corresponding results reveal the better performance of the proposed estimation algorithm over conventional ones.

**Figure 8:**
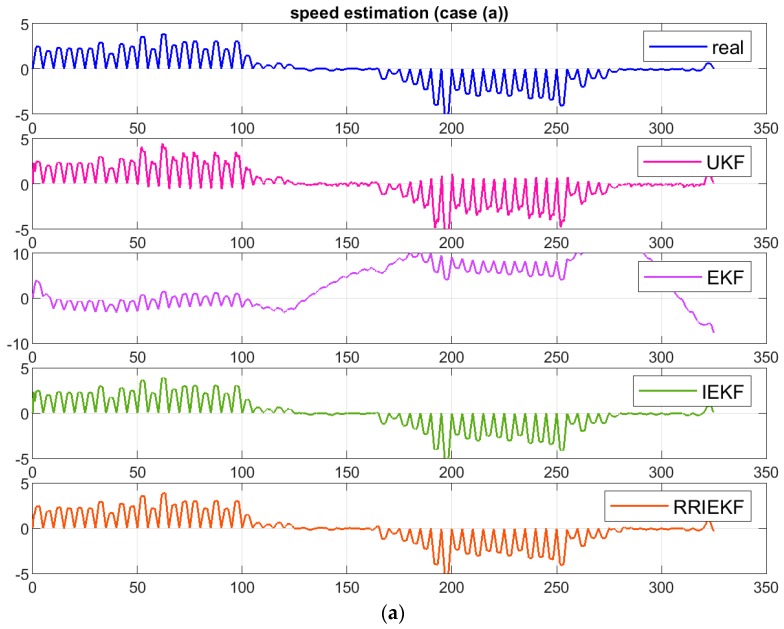

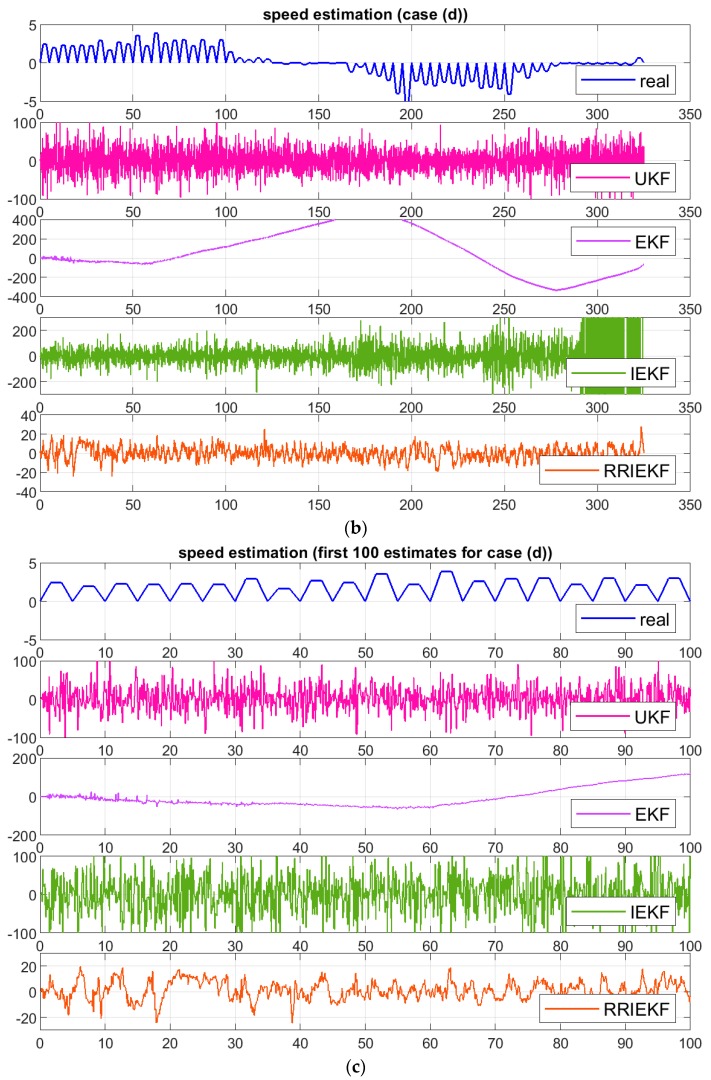
(**a**) Estimated speed along x axis for case (a), (**b**) Estimated speed along x axis for case (d). (**c**) First 100 estimates for case (d).

In Figure 8a,b, estimated speed along the x-axis is shown for study cases a and d. In Figure 8c, first 100 estimates for study case d are shown for closer and better view of estimation performance. 

The estimation performance of the proposed algorithm is significantly influenced by the presence of observations noise and measurement outlier in comparison to results in Figure 8a. However, the effect is very small comparative to other estimation algorithms. 

## 6. Conclusions and Future Perspectives

A novel algorithm is presented in this paper for estimation of non-linear and dynamic system states. Non-linear system state approximation and tracking over time is a contiguous problem, especially in navigation and localization systems, and has been addressed by several researchers. The proposed estimation algorithm is derived from the concept of robust regression theory and the Kalman filter. A weighting scheme is used to reduce the cumulative effect of outlying and noisy observations through down-weighting in an iterative process. For performance assessment, extensive numerical simulations and real tests are conducted and results are compared with the state-of-the-art estimation algorithms, EKF, UKF and IEKF. Numerical simulations demonstrate the higher approximation performance of the proposed estimator over conventional estimators, especially in tracking of dynamic states of complex model-based non-linear systems. On the other hand, the proposed algorithm is proved to be a promising process for integration of multirate sensor measurements in the presence of noise and outlying observations. In the near future, we are planning to use the proposed algorithm for localization through integration of global satellite navigation system (GNSS), IMU and visual sensor measurements and comparative analysis with proposed state of the art estimation techniques. 

## Figures and Tables

**Table 1:** Types of outliers in state estimation.

Type	Source	Effected States
Structural	Modeling	Prediction/update
Measurements	Measurement	Observations
Innovation	Estimation	Prediction

**Table 2:** Estimation Errors.

Filter	RMSE
EKF	0.106
IEKF	0.0352

**Table 3:** Estimation errors for different filters.

	Filter	UKF	EKF	IEKF	RRIEKF
wgn (dBW)					
1		0.4059	1.2714	0.4059	0.0970
5		0.9764	1.5337	0.9764	0.2149
10		2.7077	3.0297	2.4316	0.7315
20		13.7304	30.0964	13.7304	3.3963

**Table 4:** RMS estimation errors for the non-linear system model.

	Filter	UKF	EKF	IEKF	RRIEKF
wgn (dBW)					
0		7.3775	12.2015	8.5592	3.3909
5		7.0173	12.1707	8.9329	4.1361
10		8.0283	15.1359	10.3455	5.7538
20		13.4886	21.8313	17.8355	9.3390

**Table 5:** Sensor observation study cases.

a	IMU (calibrated), UWB (calibrated)
b	IMU (noise power 20dBW), UWB (calibrated)
c	IMU (calibrated), UWB (noise power 20dBW)
d	IMU (noise power 20dBW), UWB (noise power 20dBW + 1 outlier)

**Table 6:** RMSE Position Errors.

	Filter	UKF	EKF	IEKF	RRIEKF
Figure 7					
a		0.1068	0.2068	0.1071	0.1059
b		0.1069	2.4492	0.1349	0.1060
c		26.2116	36.3330	23.5349	13.6984
d		34.2215	62.8323	30.3357	14.7949

## Appendix A

**Table A1:** Relative efficiency vs. breakdown points.

BDP (%)	Efficiency (%)	α	K
50	28.7	1.547	0.1995
45	37.0	1.756	0.2312
40	46.2	1.988	0.2634
35	56.0	2.251	0.2957
30	66.1	2.560	0.3278
25	75.9	2.937	0.3593
20	84.7	3.420	0.3899
15	91.7	4.096	0.4194
0.1	96.6	5.182	0.4475
